# Machine learning-based prediction of symptomatic intracerebral hemorrhage after intravenous thrombolysis for stroke: a large multicenter study

**DOI:** 10.3389/fneur.2023.1247492

**Published:** 2023-10-20

**Authors:** Rui Wen, Miaoran Wang, Wei Bian, Haoyue Zhu, Ying Xiao, Qian He, Yu Wang, Xiaoqing Liu, Yangdi Shi, Zhe Hong, Bing Xu

**Affiliations:** ^1^Shenyang Tenth People’s Hospital, Shenyang, China; ^2^Affiliated Central Hospital of Shenyang Medical College, Shenyang Medical College, Shenyang, China; ^3^Shenyang First People’s Hospital, Shenyang Medical College, Shenyang, China

**Keywords:** machine learning, prediction, symptomatic intracerebral hemorrhage, intravenous thrombolysis, stroke, multicenter

## Abstract

**Background:**

This study aimed to compare the performance of different machine learning models in predicting symptomatic intracranial hemorrhage (sICH) after thrombolysis treatment for ischemic stroke.

**Methods:**

This multicenter study utilized the Shenyang Stroke Emergency Map database, comprising 8,924 acute ischemic stroke patients from 29 comprehensive hospitals who underwent thrombolysis between January 2019 and December 2021. An independent testing cohort was further established, including 1,921 patients from the First People’s Hospital of Shenyang. The structured dataset encompassed 15 variables, including clinical and therapeutic metrics. The primary outcome was the sICH occurrence post-thrombolysis. Models were developed using an 80/20 split for training and internal validation. Performance was assessed using machine learning classifiers, including logistic regression with lasso regularization, support vector machine (SVM), random forest, gradient-boosted decision tree (GBDT), and multilayer perceptron (MLP). The model boasting the highest area under the curve (AUC) was specifically employed to highlight feature importance.

**Results:**

Baseline characteristics were compared between the training cohort (*n* = 6,369) and the external validation cohort (*n* = 1,921), with the sICH incidence being slightly higher in the training cohort (1.6%) compared to the validation cohort (1.1%). Among the evaluated models, the logistic regression with lasso regularization achieved the highest AUC of 0.87 (95% confidence interval [CI]: 0.79–0.95; *p* < 0.001), followed by the MLP model with an AUC of 0.766 (95% CI: 0.637–0.894; *p* = 0.04). The reference model and SVM showed AUCs of 0.575 and 0.582, respectively, while the random forest and GBDT models performed less optimally with AUCs of 0.536 and 0.436, respectively. Decision curve analysis revealed net benefits primarily for the SVM and MLP models. Feature importance from the logistic regression model emphasized anticoagulation therapy as the most significant negative predictor (coefficient: −2.0833) and recombinant tissue plasminogen activator as the principal positive predictor (coefficient: 0.5082).

**Conclusion:**

After a comprehensive evaluation, the MLP model is recommended due to its superior ability to predict the risk of symptomatic hemorrhage post-thrombolysis in ischemic stroke patients. Based on decision curve analysis, the MLP-based model was chosen and demonstrated enhanced discriminative ability compared to the reference. This model serves as a valuable tool for clinicians, aiding in treatment planning and ensuring more precise forecasting of patient outcomes.

## Introduction

Symptomatic intracerebral hemorrhage (sICH) represents an infrequent yet exceptionally dreaded complication following intravenous thrombolysis for ischemic stroke. The capacity to accurately pinpoint individual patients with an elevated risk of sICH has considerable clinical ramifications, extending from aiding clinicians in therapeutic deliberations and enlightening patients and relatives regarding prognosis to tailoring monitoring regimes.

An assortment of prognostic instruments has been conceived to ascertain the risk of sICH subsequent to intravenous thrombolysis for stroke (1–5). Nonetheless, a mere handful of these models have been formulated or externally corroborated in patients undergoing endovascular treatment (EVT) for ischemic stroke (3, 5). Before a prediction model can be assimilated into clinical practice, it warrants a rigorous appraisal, with external validation serving as a crucial stage to assess its broad applicability (6). Regardless, the aggregate accuracy of these scores persistently remains moderate, highlighting a persistent demand for individualized patient management strategies.

Machine learning techniques, by virtue of their capability to apply computational algorithms to expansive datasets with manifold, multidimensional variables, may be poised to address certain shortcomings of the contemporary analytical strategies for risk prediction (7). Through capturing high-dimensional, non-linear correlations among clinical attributes, these methods could potentially enhance the precision of outcome predictions. Indeed, machine learning methodologies have commanded interest owing to their superior predictive prowess compared to traditional approaches across diverse settings and disease states (8–10). However, to the best of our knowledge, there is a conspicuous dearth of research employing machine learning models trained on large-scale, multicenter data to predict sICH.

Furthermore, the majority of risk models scrutinized hitherto have predominantly been developed and validated in patients of European American lineage, thus creating a lacuna in our comprehension of the risk in patients from diverse racial backgrounds.

Given this scenario, our study endeavors to bridge these gaps by analyzing data accrued from multiple centers across China to devise machine learning-based triage models, predicting the likelihood of sICH-ensuing intravenous thrombolysis. We postulate that these models will supersede traditional risk prediction models in terms of precision and adaptability across heterogeneous patient cohorts. Additionally, we intend to externally validate the predictive competency of our models in patients treated with intravenous thrombolysis in everyday clinical practice. We aspire that our research will culminate in the enhancement of management strategies for patients susceptible to sICH following an ischemic stroke.

## Methods

This study is designed as an observational, multicenter, retrospective cohort study, encompassing data from several comprehensive hospitals. It primarily aims to evaluate and compare the efficacy of different machine learning models in predicting outcomes for ischemic stroke patients after thrombolysis treatment. To ensure ethical considerations and maintain research integrity, this study received formal approval from the Research Ethics Committee of Shenyang First Hospital (Approval Number: 2023SYKYPZ08).

### Datasets

We initiated our research drawing from the Shenyang Stroke Emergency Map database, which caters to a vast population exceeding 9 million and stands as a keystone for the citywide initiative aiming at the enhancement of stroke care quality. Specialized personnel from 30 diverse and comprehensive hospitals directly uploaded clinical data to this database. With this extensive data pool, our focus was on a distinct cohort of 8,924 acute ischemic stroke (AIS) patients who underwent thrombolytic therapy. These patients were gathered from 29 of these hospitals, and our study spanned from January 2019 to December 2021. Our primary inclusion criteria were patients aged 18 years or older who were confirmed as having an ischemic stroke upon hospitalization. First, we excluded patients who had undergone EVT, as recorded in the database. Furthermore, patients missing data on either their admission National Institutes of Health Stroke Scale (NIHSS) score or post-thrombolysis NIHSS score were removed. Moreover, a crucial aspect of our data filtering process involved omitting patients who lacked information simultaneously in both the Swallowing Function Score and the Admission mRS Score columns. Given the clinical importance of these metrics in evaluating patient health and treatment outcomes, their absence could lead to a potentially incomplete or misleading patient assessment. Beyond these specific data-related exclusion criteria, we also excluded patients with severe organ dysfunctions, such as heart, liver, and kidney issues, as well as those with malignant tumors or other significant infections. Additionally, cases with other missing key feature data or those with poor data quality were also disregarded.

Subsequently, an additional independent testing cohort was established, comprising 2,046 consecutive patients who received thrombolytic therapy at the First People’s Hospital of Shenyang during the identical timeframe. Using the same stringent inclusion and exclusion criteria as our primary cohort, 1,921 patients from this independent group were finalized to serve as the testing set for our study.

Following the stringent patient selection criteria, our data preparation encountered an anticipated challenge: missing values. Within the scope of our study, absent data for both predictor and outcome variables were evident. Such omissions may be attributed to various reasons, such as unperformed tests or incomplete patient records. To address this intricacy, we adopted the Multiple Imputation by Chained Equations (MICE) approach, employing the mice package in R (11). During the imputation process, we incorporated all predictor variables and the outcome variable, opting for the random forest (RF) method owing to its adeptness in deciphering intricate data patterns. It is noteworthy that, despite the inherent complexity of our dataset, we abstained from introducing interaction terms in the imputation model.

We proceeded with the creation of 20 imputed datasets. For the purpose of aggregation, the imputed values across these datasets were averaged with categorical variables determined by the model. Post-imputation, a thorough analysis of each dataset ensued. To consolidate the findings, Rubin’s rules (12) were meticulously applied, yielding a harmonized output. To corroborate the robustness of our imputation technique, a sensitivity analysis juxtaposing the imputed and original datasets was performed, the details of which have been annexed.

### Predictors

The structured dataset encapsulated 15 variables. This included the following 12 clinical metrics: gender, age, postawakening stroke, in-hospital stroke, body mass index (BMI), systolic blood pressure (SBP), diastolic blood pressure (DBP), Admission mRS Score, Admission NIHSS Score, Swallowing Function Score, onset-to-needle time (ONT), and TOAST Classification. Additionally, there were three therapeutic metrics: thrombolytic drugs, antiplatelet therapy, and anticoagulation therapy.

### Primary outcomes

The primary outcome was the occurrence of sICH following thrombolysis. sICH was defined as “any neurological deterioration (increase of NIHSS≥1) within 36 h after tPA administration that is attributed to intracerebral hemorrhage (ICH) confirmed by CT or MRI,” as per the definition of the National Institute of Neurological Disorders and Stroke (NINDS) (13).

### Model development and validation

The training cohort was arbitrarily divided into two subsets: 80% for model training and 20% for internal validation. Subsequently, experiments with five machine learning classifiers—logistic regression with lasso regularization (lasso regression), support vector machine (SVM), RF, gradient-boosted decision tree (GBDT), multilayer perceptron (MLP)—were implemented to generate our proprietary models for the prediction of each study outcome. The reference model employed a logistic regression with no regularization and was trained using the “saga” solver. An exhaustive grid search was applied to optimize the hyperparameters for these classifiers within predefined ranges. The internal validation cohort was specifically employed to adjust the models’ parameters. Through the grid search method, we identified the optimal hyperparameters corresponding to the highest AUC value on the internal validation set for each model. In the training cohort (80% randomly selected samples), we constructed a reference model and five proprietary machine learning models for each outcome.

Model performance was appraised in the external validation cohort according to a spectrum of learning metrics (mean area under the receiver operating characteristic curve [AUC] and decision curve analysis [DCA]), and the optimal performing model for the study outcome was selected. The DCA is a measure that considers the varying weights of different misclassification types with a direct clinical interpretation (for example, trade-offs between undertriage and overtriage for each model) (14, 15). Specifically, the relative effect of false-negative (undertriage) and false-positive (overtriage) results, given a threshold probability (or clinical preference), was calculated to produce a net benefit in each model. The net benefit of each model over a specified range of threshold probabilities of outcome was graphically demonstrated as a decision curve.

### Data imbalance

In the dataset, the sICH prevalence was significantly low, at approximately 1.6%, leading to a noticeable class disparity. To mitigate this imbalance and enhance model performance, we utilized the Synthetic Minority Over-sampling Technique (SMOTE). SMOTE works by generating synthetic instances for the minority class, drawing upon the characteristics of existing samples. By using this technique, we equilibrated the representation of the sICH positive and majority classes, ensuring a more sensitive and accurate model for identifying potential sICH cases.

### Feature importance

The model exhibiting the highest AUC was chosen to highlight the importance of features, ensuring insights into the most influential predictors.

### Statistical analysis

Categorical variables are delineated as count (%) and continuous variables as mean (SD) or median (interquartile range). The presence of a normal distribution was confirmed by the Kolmogorov–Smirnov test. We employed the *t*-test to evaluate disparities between parametric continuous variables, the Mann–Whitney *U* test for non-parametric variables, the *χ*^2^ test for categorical variables, and the Fisher’s exact test for 2 × 2 tables. No correction for multiple testing was instituted. A two-sided value of *p* of <0.05 was deemed statistically significant. All analyses were conducted with R version 4.1.2 and Python version 3.10.2.

## Results

Table 1 illustrates the baseline characteristics of patients from the training cohort (*n* = 6,369) and the external validation cohort (*n* = 1,921). Both groups showed a similar median age of 65 years and a gender distribution where women accounted for approximately 30%. Notably, there was a significant difference in in-hospital stroke rates, with the training cohort having 5.2% compared to 1.5% in the external validation cohort. Variations were also observed in clinical metrics such as SBP, DBP, Swallowing Function Score, and TOAST Classification. The use of recombinant tissue plasminogen activator (rt-PA) was predominant in the training cohort (89%), while urokinase was more frequently used in the validation cohort (35%). The sICH incidence was slightly higher in the training cohort at 1.6% compared to the validation cohort’s 1.1%. Overall, while some demographic characteristics aligned between the cohorts, clinically relevant variations highlight the significance of external validation in model assessments.

**Table 1:** Baseline features of included cohorts.

Characteristic	Training cohort (*N* = 6,369)	External validation cohort (*N* = 1,921)	*p*-value
Gender			0.7
Male	4,507 (71%)	1,351 (70%)	
Female	1,859 (29%)	570 (30%)	
Unknown	3	0	
Age, median (Q1, Q3), years	65 (57, 71)	65 (58, 72)	0.034
Unknown	3	0	
Postawakening stroke			0.2
No	6,210 (98%)	1,882 (98%)	
Yes	159 (2.5%)	39 (2.0%)	
In-hospital stroke			<0.001
No	6,039 (95%)	1,892 (98%)	
Yes	330 (5.2%)	29 (1.5%)	
BMI	24.2 (21.9, 26.3)	24.1 (22.1, 26.1)	0.9
Unknown	440	37	
SBP (Q1, Q3), mmHg	157 (141, 169)	149 (138, 160)	<0.001
DBP (Q1, Q3), mmHg	90 (80, 97)	85 (79, 93)	<0.001
Admission mRS score			
0	3,271 (53%)	546 (28%)	
1	845 (14%)	386 (20%)	
2	487 (7.9%)	535 (28%)	
3	416 (6.7%)	195 (10%)	
4	925 (15%)	191 (9.9%)	
5	216 (3.5%)	67 (3.5%)	
6	15 (0.2%)	1 (<0.1%)	
Unknown	194	0	
Admission NIHSS score	6 (4, 11)	3 (2, 6)	<0.001
Swallowing function score			<0.001
1	2,286 (45%)	1,131 (93%)	
2	1,706 (34%)	82 (6.7%)	
3	387 (7.7%)	1 (<0.1%)	
4	351 (7.0%)	1 (<0.1%)	
5	314 (6.2%)	1 (<0.1%)	
Unknown	1,325	705	
TOAST classification			<0.001
LAA	3,345 (53%)	1,519 (79%)	
CE	508 (8.0%)	94 (4.9%)	
SAA	2,046 (32%)	282 (15%)	
SOE	29 (0.5%)	3 (0.2%)	
SUE	441 (6.9%)	23 (1.2%)	
Thrombolytic drug			<0.001
rt-PA	5,653 (89%)	1,211 (63%)	
Urokinase	634 (10.0%)	663 (35%)	
Others	82 (1.3%)	47 (2.4%)	
ONT, Median (Q1, Q3), min	170 (127, 227)	158 (113, 222)	<0.001
Unknown	32	0	
Antiplatelet therapy			<0.001
No	688 (11%)	112 (5.8%)	
Yes	5,437 (89%)	1,809 (94%)	
Unknown	244	0	
Anticoagulation therapy			<0.001
No	5,730 (95%)	1,890 (99%)	
Yes	318 (5.3%)	28 (1.5%)	
Unknown	321	3	
sICH, *n* (%)	99 (1.6%)	22 (1.1%)	0.2

BMI, body mass index; SBP, systolic blood pressure; DBP, diastolic blood pressure; LAA, large-artery atherosclerosis stroke; CE, cardioembolic stroke; SAA, small artery occlusion or lacunar stroke; SOE, stroke of other determined etiology; SUE, stroke of undetermined etiology; ONT, onset-to-needle time; sICH, symptomatic intracerebral hemorrhage.

**Figure 1:**
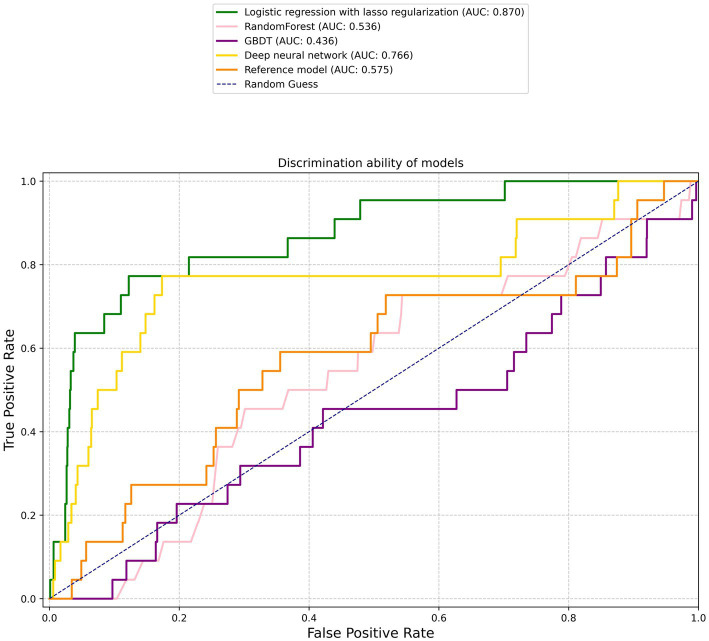
Receiver operating characteristic curves. The corresponding values of the area under the curve for each model are presented in Table 2.

Figure 1 graphically depicts the discriminative capacities of various models using their receiver operating characteristic curves. The reference model exhibited a rather subdued performance, registering a C statistic of 0.575 (95% confidence interval [CI], 0.44–0.71). Surprisingly, not all machine learning models outperformed the reference. For instance, the RF and GBDT models manifested subpar performances with C statistics of 0.536 (95% CI, 0.42–0.653) and 0.436 (95% CI, 0.305–0.568), respectively (Table 2).

**Table 2:** Predictive ability of the reference model and five machine learning models for sICH in patients with ischemic stroke who received thrombolysis.

Model	AUC with CI	*P* value	Sensitivity (95% CI)	Specificity (95% CI)	PPV (95% CI)	NPV (95% CI)	PLR (95% CI)	NLR (95% CI)
Reference model	0.575 (0.44–0.71)	[Reference]	0.561 (0.404–0.68)	0.663 (0.022–0.825)	0.019 (0.012–0.028)	0.992 (0.989–1.0)	1.659 (1.022–2.457)	0.661 (0.0–0.92)
Logistic regression with lasso regularization	0.87 (0.79–0.95)	<0.001	0.781 (0.727–0.818)	0.876 (0.813–0.918)	0.657 (0.607–0.694)	0.798 (0.755–0.829)	0.211 (0.169–0.250)	0.111 (0.072–0.150)
Random forest	0.536 (0.42–0.653)	0.712	0.662 (0.500–0.773)	0.533 (0.400–0.687)	0.195 (0.145–0.258)	0.625 (0.529–0.668)	0.042 (0.031–0.057)	0.010 (0.006–0.016)
GBDT	0.436 (0.305–0.568)	0.235	0.399 (0.045–0.911)	0.674 (0.100–0.988)	0.072 (0.007–0.160)	0.364 (0.056–0.605)	0.021 (0.004–0.052)	0.009 (0.000–0.040)
MLP	0.766 (0.637–0.894)	0.04	0.701 (0.591–0.818)	0.839 (0.707–0.927)	0.540 (0.451–0.619)	0.723 (0.631–0.803)	0.161 (0.111–0.222)	0.078 (0.036–0.140)
SVM	0.582 (0.472–0.692)	0.94	0.664 (0.364–0.818)	0.612 (0.487–0.830)	0.277 (0.182–0.375)	0.644 (0.411–0.744)	0.061 (0.043–0.082)	0.017 (0.011–0.030)

However, two machine learning models notably stood out: the logistic regression with lasso regularization and the MLP. The former displayed a prominent C statistic of 0.87 (95% CI, 0.79–0.95; *p* < 0.001), while the latter exhibited a C statistic of 0.766 (95% CI, 0.637–0.894; *p* = 0.04). When pitted against each other, the difference in AUC between the two was borderline significant at a value of *p* of 0.058, suggesting a potential edge for the logistic regression model, albeit not definitively so.

**Figure 2:**
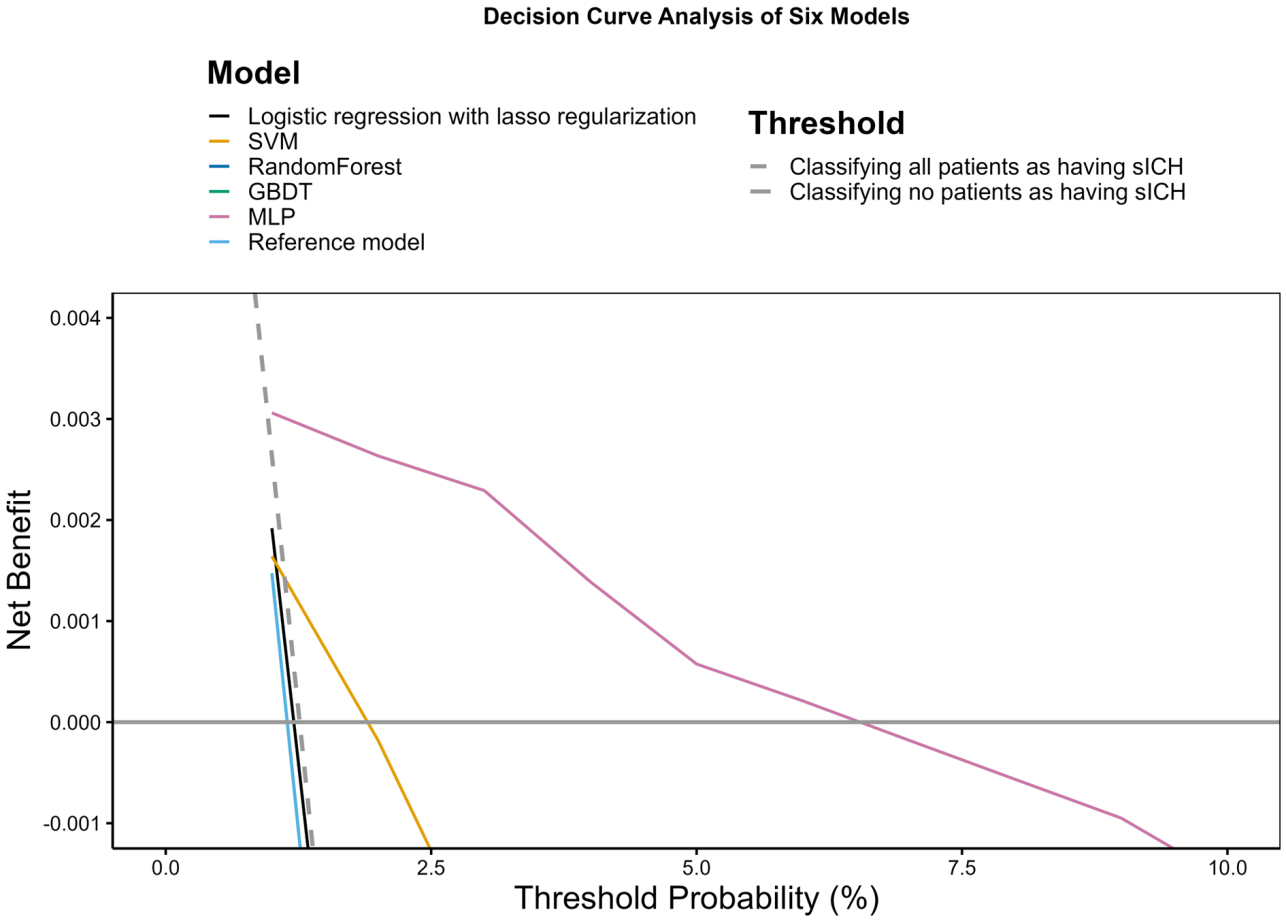
Decision curve analysis. The *x*-axis indicates the threshold probability for hospitalization outcome. The *y*-axis indicates the net benefit. The curves (decision curves) indicate the net benefit of models (the reference model and five machine learning models) as well as two clinical alternatives (classifying no patients as having sICH vs. classifying all patients as having sICH) over a specified range of threshold probabilities of outcome. Only the SVM and MLP models exhibited a positive net benefit.

According to the DCA shown in Figure 2, only the SVM and MLP models demonstrated a net benefit. Notably, the MLP model displayed a broader range of threshold probabilities where it had a net benefit, outperforming other models. The remaining models did not exhibit discernible net benefits.

**Figure 3:**
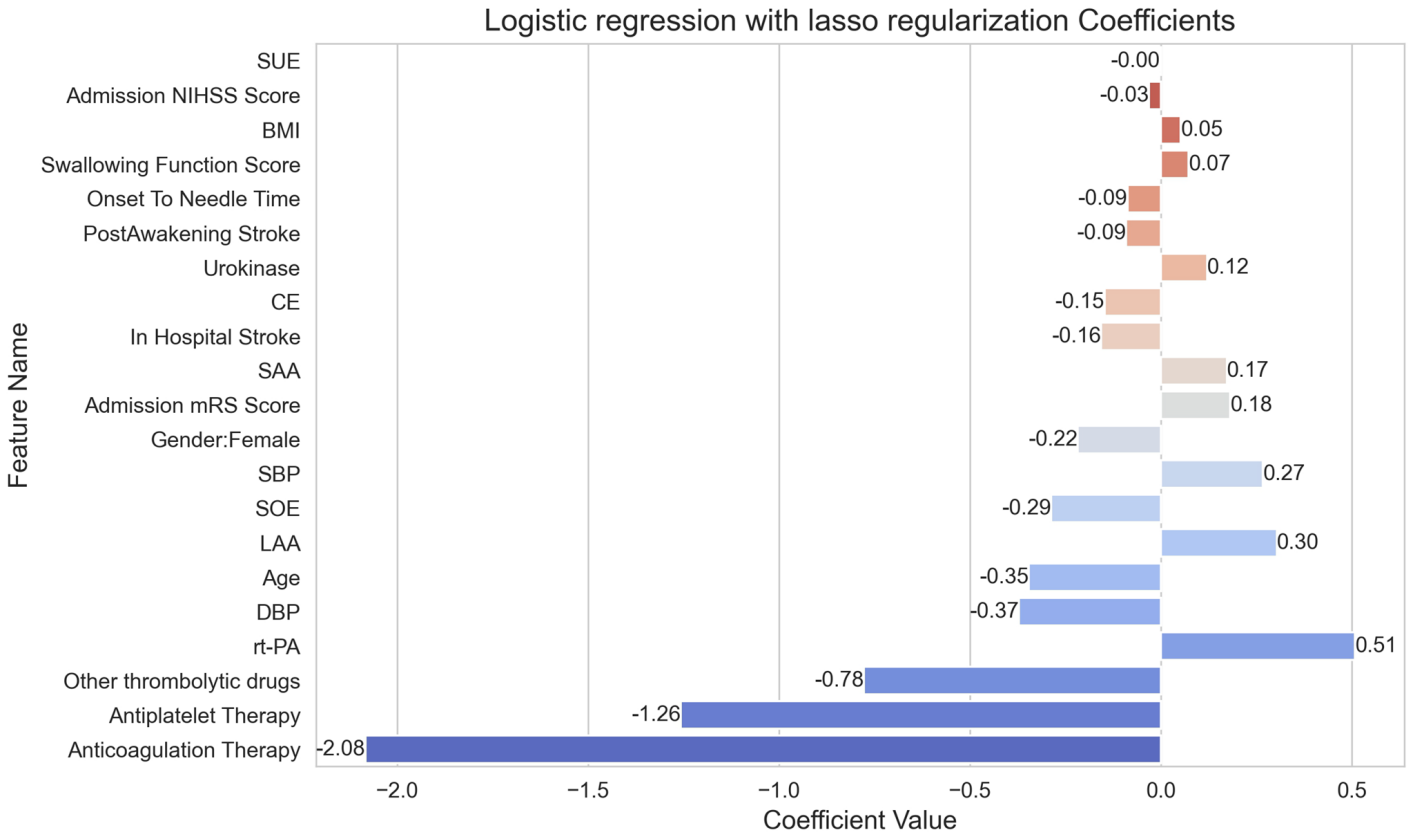
Importance of features as determined by logistic regression with lasso regularization.

In Figure 3, the importance of various features is depicted through their coefficients from the logistic regression with lasso regularization model. This model was specifically chosen for illustrating feature importance due to its highest AUC value, showcasing its superior discriminative capability among the evaluated models.

Anticoagulation therapy emerged as having the most pronounced negative effect on the outcome, as indicated by its coefficient of −2.0833, suggesting a decreasing likelihood of the outcome with an increase in this variable. On the contrary, rt-PA stands out as the feature having the most positive influence on the outcome, bearing a coefficient of 0.5082. Other influential positive predictors include TOAST Classification 1, SBP, and Admission mRS Score, with coefficients of 0.3033, 0.2668, and 0.1811, respectively. Meanwhile, features such as age and DBP present as detractors from the outcome due to their negative coefficients of −0.3456 and −0.3713, respectively. Interestingly, certain features such as TOAST Classification 4 exhibited negligible influence on the outcome, as signified by their coefficients hovering close to zero. This visualization aids in elucidating the varying extents to which different predictors influence the outcome, as interpreted from the logistic regression model.

## Discussion

In this study, we pinpointed readily available clinical and laboratory factors prior to thrombolysis and validated the effectiveness of machine learning techniques in forecasting sICH post-thrombolysis. The machine learning workflow was formulated solely from real-world, multicenter patient databases. To the best of our understanding, this constitutes the most comprehensive multicenter study to develop and assess machine learning models with an unparalleled sample size and to subsequently examine their clinical applicability in independent datasets.

While the logistic regression with lasso regularization demonstrated a marginally higher AUC value compared to the MLP, a DeLong test comparison between the AUCs of both models yielded a value of *p* greater than 0.05, suggesting no statistically significant difference. More importantly, in the DCA evaluation, the MLP showcased superior performance over the logistic regression model with lasso regularization. This reinforces our recommendation of the MLP, emphasizing its potential for enhanced clinical applicability and overall patient benefit.

Our machine learning models achieved prediction accuracies exceeding 80% on the validation set, with a peak accuracy of 87%, thereby showcasing commendable predictive performance in practice when contrasted with the reference model. Compared to conventional models, machine learning models showed superior performance in forecasting sICH post-thrombolysis. Although a previous study utilized multicenter data for modeling and external validation, it was somewhat limited, only incorporating 136 cases in the external validation set to evaluate the model’s predictive performance in scenarios of low sICH incidence in real-world settings, which may not accurately reflect the model’s performance (16). These machine learning models also attained higher sensitivity and specificity in predicting sICH outcomes.

Moreover, our research utilized clinical decision curves (CDCs) to evaluate the model’s performance, offering a visual depiction of the sensitivity and specificity trade-off across varied threshold probabilities. CDCs are instrumental in identifying the optimal threshold for clinical decision-making and pinpointing the patient population where the model exhibits maximum effectiveness. They provide a succinct method of interpreting and applying prediction models and serve as a crucial tool in guiding clinical decision-making, particularly in scenarios that involve multiple prediction models or clinical strategies (15). The net benefit was also more substantial across a wide range of threshold probabilities in machine learning approaches.

Utilizing logistic regression with lasso regularization, we pinpointed anticoagulation therapy, antiplatelet therapy, other thrombolytic drugs, rt-PA, DBP, age, LAA, SUE, SBP, gender, and Admission mRS Score as significant predictors for the occurrence of sICH post-thrombolysis in patients. Notably, our identification of these predictors aligns well with the broader literature. Hypertension (17), diabetes (18), older age (19), higher body mass index (20), and cardioembolic stroke (CE) (5) emerged as the primary risk factors for hemorrhage in patients. This is further corroborated by previous studies that recognized hypertension, elevated blood pressure, and older age as risk factors for post-thrombolysis hemorrhage (21, 22).

In the realm of our research, which primarily focuses on prediction, we identified antiplatelet therapy, anticoagulation therapy, age, and gender as significant predictors of sICH. Notably, the female gender was negatively associated with increased sICH incidence. This finding aligns with certain past studies that have identified a similar protective attribute associated with female gender in the context of sICH (23). However, our results diverge from the conventional understanding where anticoagulation (24) and antiplatelet (25, 26) therapies have been historically linked with heightened hemorrhagic risks post-thrombolysis and the frequent association of older age (27) with sICH. However, it is pivotal to understand that our aim differed from traditional studies, as we were more oriented toward forecasting outcomes rather than dissecting risk factors. Thus, while we delineate the coefficients for these predictors, the directional values, especially in the absence of associated *p*-values, remain interpretative. They signify predictive relationships in our dataset, not necessarily causal connections. Such distinctions become essential when weighing our predictive findings against traditional studies aiming primarily at risk factor exploration. Moreover, variations in patient selection, treatments employed, dosages, and timing across studies could influence these disparities. Our data’s alternative trend emphasizes the critical need to consider the myriad of concurrent factors or co-morbidities potentially interacting with these predictors.

Studies have indicated that the onset of sICH is among the most severe complications post-thrombolysis, a typical treatment for ischemic stroke (28). Accurate sICH prediction can aid clinicians in formulating appropriate treatment strategies and enhancing patient outcomes. Traditional sICH prediction models bear limitations regarding accuracy and dependability due to the intricate interplay of numerous clinical and imaging factors. Hence, the application of machine learning models for predicting sICH in patients with AIS has garnered growing interest recently, given its potential clinical significance. While adding a larger set of predictive factors such as comprehensive disease history, continuous vital sign monitoring, and imaging data might enhance the model’s capabilities, acquiring such a vast array of predictors in practice is challenging due to constraints related to information, resources, and time, especially in multicenter datasets. An alternative strategy to aid clinical decision-making in predicting post-thrombolysis sICH involves the deployment of contemporary machine learning methods to navigate the complex non-linear interactions among predictive factors (6).

Recent investigations have illuminated the potential of machine learning models in predicting various clinical outcomes, such as hemorrhagic transformation post-ischemic stroke (29), diabetic retinopathy (30), and small-bowel diseases *via* capsule endoscopy (31). These models are developed by leveraging intricate non-linear relationships between predictors and outcomes in extensive datasets, enabling the identification of clinically relevant predictors and enhancing prediction accuracy. Our study expands on previous reports, showcasing the superior capacity of modern machine learning methods in predicting clinical outcomes and guiding management through modeling and external validation with a large sample size of nearly 10,000 patients from 30 hospitals.

However, our study has some limitations. The study sample was primarily from hospitals in the northeast region of China, so our findings need to be validated and generalized to other regions to ensure their universality and applicability. Future studies should also expand the sample size to further validate our findings. While our machine learning algorithms exhibited commendable predictive acumen for sICH, they remain susceptible to the restrictions imposed by currently accessible data and may not invariably furnish accurate prognostications for individual patient outcomes. However, it certainly underscores the feasibility of a machine-learning model for crafting personalized risk prognostication. To mitigate these concerns, we advocate further exploration and enhancement of data dissemination and transparency. It merits highlighting that our investigation incorporated a circumscribed set of variables for the machine learning paradigms, and the inclusion of additional germane variables may bolster the model’s performance.

Notwithstanding these constraints, the creation of triage models predicated on machine learning principles continues to be a promising prospect for ameliorating the prognosis of stroke patients and augmenting clinical decision-making within the framework of thrombolytic treatment. Prospective studies should endeavor to tackle these constraints and unearth methods to refine these models further for optimal clinical utility. With the integration of a more expansive set of variables and ongoing refinement, these models could evolve into formidable instruments for clinicians managing AIS patients. By addressing these constraints, we can persistently enhance these models’ precision and applicability, culminating in improved patient outcomes and refined clinical decision-making.

## Conclusion

To summarize, our study highlights the efficacy of the MLP as a machine learning technique in prognosticating the risk of symptomatic hemorrhage following thrombolysis in patients with ischemic stroke. Based on DCA, the MLP was chosen, illuminating its strength as a predictive tool. Through this approach, we have been able to elucidate critical predictive determinants of hemorrhagic risk. Despite potential limitations, the MLP-based model presented here stands as a potent instrument for clinicians, offering insights into treatment planning and enabling more accurate forecasts of patient outcomes.

## Data availability statement

The raw data supporting the conclusions of this article will be made available by the authors, without undue reservation.

## Ethics statement

The studies involving humans were approved by the Research Ethics Committee of Shenyang First Hospital. The studies were conducted in accordance with the local legislation and institutional requirements. Written informed consent for participation was not required from the participants or the participants’ legal guardians/next of kin in accordance with the national legislation and institutional requirements.

## Author contributions

RW wrote the main manuscript text and prepared all figures. MW, WB, HZ, YX, QH, YW, XL, YS, and ZH contributed to this work by providing resources and acquiring the data necessary for the research. BX provided oversight, direction for the project, reviewed, and edited the final manuscript. All authors reviewed and approved the final manuscript.
