# Supplementary material for: Machine learning-based prediction of symptomatic intracerebral hemorrhage after intravenous thrombolysis for stroke: a large multicenter study
Source: Front Neurol. 2023 Oct 20;14:1247492. doi: 10.3389/fneur.2023.1247492 (PMC10624225; doi:10.3389/fneur.2023.1247492)
Supplement: Supplementary file 1 [file Data_Sheet_1.PDF]

The specific ranges for each hyperparameter:

```
param_grids = {
    'LogisticRegression': {
        'C': [0.1, 0.5, 1, 5, 10, 50, 100,150]    # Added 0.5 and 5 for finer granularity.
    },
    'SVC': {
        'C': [0.1, 1, 10],
        'kernel': ['linear', 'rbf'],
        'gamma': ['scale', 'auto'],
    },
    'RandomForest': {
        'n_estimators': [100, 200, 300, 400],    # Extended to 300 and 400
        'max_depth': [20, 30, 40, 50],    # Extended to 40 and 50
        'min_samples_split': [2, 4, 6, 10],    # Added 4 and 6 for finer granularity.
        'min_samples_leaf': [1, 2, 3, 4]    # Added 3 for finer granularity.
    },
     'GBDT': {
        'n_estimators': [100, 200, 300, 400],
        'learning_rate': [0.05, 0.1, 0.15],
        'max_depth': [3, 4, 5, 6],
        'max_leaf_nodes': [2, 3, 4, 5],
        'loss': ['log_loss', 'exponential'],
        'min_samples_leaf': [10, 20, 30, 40]
    },
    'MLP': {
        'hidden_layer_sizes': [(50,), (100,), (100, 100), (150, 150)],    # Added (50,) and (150, 150) for finer granularity and extension.
        'activation': ['tanh', 'relu'],
        'alpha': [0.001, 0.01, 0.05, 0.1, 0.5]    # Added 0.05 and 0.5 for finer granularity.
    }
}
```

The final selected values:

Method	Best Params
Logistic regression with lasso regularization	{'C': 10}
SVM	{'C': 10, 'kernel': 'rbf', 'gamma': 'scale'}
RandomForest	{'n_estimators': 200, 'max_depth': 40, 'min_samples_split': 2, 'min_samples_leaf': 1}

GBDT	{'n_estimators': 400, 'learning_rate': 0.15, 'max_depth': 4, 'max_leaf_nodes': 5, 'loss': 'log_loss', 'min_samples_leaf': 20}
MLP	{'hidden_layer_sizes': (150, 150), 'activation': 'tanh', 'alpha': 0.001}
