# Supplementary material for: Machine learning-based prediction of symptomatic intracerebral hemorrhage after intravenous thrombolysis for stroke: a large multicenter study
Source: Front Neurol. 2023 Oct 20;14:1247492. doi: 10.3389/fneur.2023.1247492 (PMC10624225; doi:10.3389/fneur.2023.1247492)
Supplement: Supplementary file 2 [file Data_Sheet_2.PDF]

Logistic Regression:

Training AUC: 0.9186

Validation AUC: 0.9125

Difference: 0.0061

This suggests that the Logistic Regression model has comparable performance on both the training and validation sets, indicating its commendable generalization capabilities.

SVC:

Training AUC: 0.9978

Validation AUC: 0.9952

Difference: 0.0026

Although the SVC model exhibited near-perfect performance on the training set, it also performed impressively on the validation set.

Random Forest:

Training AUC: 1.0000

Validation AUC: 0.9998

Difference: 0.0002

The Random Forest model excelled on both datasets, suggesting that it might be fitting the data exceptionally well.

GBDT:

Training AUC: 0.9997

Validation AUC: 0.9992

Difference: 0.0005

The GBDT model also showcased stellar performance, with its results on both datasets being remarkably close.

MLP:

Training AUC: 1.0000

Validation AUC: 0.9988

Difference: 0.0012

The MLP neural network displayed perfection on the training data, and its performance on the validation data was also very akin to its training data results.

From the results presented, it's evident that all the models demonstrated closely aligned performance on the training and validation datasets. This attests to our models' robust generalization abilities, with no conspicuous signs of overfitting. We appreciate your guidance and suggestions and will diligently consider these results and amend our manuscript accordingly.
