# Supplementary material for: Machine learning-based prediction of symptomatic intracerebral hemorrhage after intravenous thrombolysis for stroke: a large multicenter study
Source: Front Neurol. 2023 Oct 20;14:1247492. doi: 10.3389/fneur.2023.1247492 (PMC10624225; doi:10.3389/fneur.2023.1247492)
Supplement: Supplementary file 3 [file Data_Sheet_3.PDF]

Appendix: Package Citations
Below are the R packages utilized during the analysis:

No.	Package Name	Purpose	Citation
1	dplyr	Data manipulation and transformation.	Hadley Wickham, et al. (2021). dplyr: A Grammar of Data Manipulation
2	visdat	Visualization of data.	Nicholas Tierney and Jennifer Bryan (2017). visdat: Preliminary Visualisation of Data
3	naniar	Missing data analysis.	Please refer to the package documentation for the official citation.
4	mice	Multiple imputation.	Stef van Buuren, et al. (2011). mice: Multivariate Imputation by Chained Equations in R
5	gtsummary	Producing descriptive statistics tables.	Please refer to the package documentation for the official citation.
6	huxtable	Creation and manipulation of table data.	Please refer to the package documentation for the official citation.
7	officer	Exporting to Word documents.	Please refer to the package documentation for the official citation.
8	tidyverse	Collection of data science related packages.	Hadley Wickham, et al. (2019). Welcome to the tidyverse
9	tidyr	Data tidying.	Hadley Wickham and Lionel Henry (2021). tidyr: Tidy Messy Data

Below are the Python packages utilized during the analysis:

No.	Package Name	Purpose
1	pandas	Data manipulation and analysis.
2	sklearn	Machine learning and data mining.
3	imblearn	Handling imbalanced datasets.
4	collections	Container datatypes (e.g., Counter).
5	itertools	Creating iterators for efficient looping.
6	joblib	Lightweight pipelining in Python.
7	numpy	Scientific computing with arrays.
8	random	Implement pseudo-random number generators.
9	time	Time-related tasks.

No.	Package Name	Purpose
10	xgboost	Gradient boosting framework.
11	lightgbm	Gradient boosting framework (designed to be distributed).
12	scipy.stats	Statistical functions and transformations.
13	seaborn	Statistical data visualization.
14	matplotlib	2D plotting library.
