# Supplementary material for: Machine learning-based prediction of symptomatic intracerebral hemorrhage after intravenous thrombolysis for stroke: a large multicenter study
Source: Front Neurol. 2023 Oct 20;14:1247492. doi: 10.3389/fneur.2023.1247492 (PMC10624225; doi:10.3389/fneur.2023.1247492)
Supplement: Supplementary file 4 [file Data_Sheet_4.PDF]

1.Analysis on Frequency and Pattern of Missing Data

(1)Methodology:

Utilized the naniar package to conduct an MCAR (Missing Completely At Random) test. Performed the MCAR test on columns with missing data in the dataset.

(2)MCAR Test Results:

Statistic: 736.

Degrees of Freedom: 147.

p-value: 0.

Number of Missing Patterns: 26.

(3)Interpretation:

A p-value of 0 indicates that we reject the hypothesis that the data is MCAR. This suggests that the missing values are not completely random.

There are 26 different missing data patterns observed.

(4)Further Action:

Given that the missing values are not completely random, relying on simple imputation methods would not be appropriate. This is the primary reason for choosing multiple imputation as a strategy to address the missing data, ensuring a more robust and accurate representation of the underlying data.

2.Sensitivity Analysis Report

To evaluate the impact of our data imputation method, we conducted a sensitivity analysis on the data before and after imputation. Here's what we found:

(1)Overall View:

Our sensitivity analysis aimed to compare the distribution of variables before and after data imputation. For most variables, the distribution post-imputation closely mirrors the original data, indicating that the imputation process did not introduce significant bias.

(2)Detailed Analysis:

Gender, Age, BMI, Admission_mRS_Score, and Onset_To_Needle_Time: The post-imputation data is highly similar to the original data. For these variables, the P-values are all above 0.9, suggesting that the imputation did not significantly alter their distribution.

Swallowing_Function_Score: The imputation process had a notable effect on the distribution of this variable. Specifically, there was a 5% increase in frequency for the score of 1. Furthermore, minor shifts were observed in other categories. The P-value is less than 0.001, indicating that special attention should be given to this variable in subsequent analyses.

Antiplatelet_Therapy and Anticoagulation_Therapy: Both variables maintained similarity with the original data post-imputation, with a P-value of 0.5.

Table 1 Comparison of Feature Distributions Between Original and Imputed Data

Characteristic	Original Data	Imputed Data	P Value
Gender			>0.9
0	4,507 (71%)	4,509 (71%)	
1	1,859 (29%)	1,860 (29%)	
Unknown	3	0	
Age	65 (57, 71)	65 (57, 71)	>0.9
Unknown	3	0	
BMI	24.2 (21.9, 26.3)	24.2 (22.0, 26.1)	>0.9
Unknown	440	0	
Admission_mRS_Score			>0.9
0	3,271 (53%)	3,426 (54%)	
1	845 (14%)	860 (14%)	
2	487 (7.9%)	490 (7.7%)	
3	416 (6.7%)	417 (6.5%)	
4	925 (15%)	944 (15%)	
5	216 (3.5%)	217 (3.4%)	
6	15 (0.2%)	15 (0.2%)	
Unknown	194	0	
Swallowing_Function_Score			<0.001
1	2,286 (45%)	3,180 (50%)	
2	1,706 (34%)	1,995 (31%)	
3	387 (7.7%)	415 (6.5%)	
4	351 (7.0%)	384 (6.0%)	
5	314 (6.2%)	395 (6.2%)	
Unknown	1,325	0	

Onset_To_Needle_Time	170 (127, 227)	170 (127, 227)	>0.9
Unknown	32	0	
Antiplatelet_Therapy			0.5
0	688 (11%)	693 (11%)	
1	5,437 (89%)	5,676 (89%)	
Unknown	244	0	
Anticoagulation_Therapy			0.5
0	5,730 (95%)	6,051 (95%)	
1	318 (5.3%)	318 (5.0%)	
Unknown	321	0	

Further Discussion on Swallowing_Function_Score:

Upon analyzing the Swallowing_Function_Score, we observed varying strength of association across some score categories between the original and imputed data.

Swallowing_Function_Score2: In the original data, the Odds Ratio (OR) stands at 1.5227 with a 95% confidence interval ranging from 0.8658 to 2.6781 and corresponds to a P-value of 0.1443, indicating a non-significant relation with the target variable. However, in the imputed data, the OR is 1.3203, with a 95% CI from 0.8299 to 2.1007, and a P-value of 0.2408, further supporting the non-significant relationship.

Swallowing_Function_Score3: In the original data, the OR is 2.8785 with a 95% CI from 1.3917 to 5.9537, and a P-value of 0.0044, showing a significant positive association. In the imputed data, although the OR drops to 2.1374, a significant association persists, as seen from the 95% CI of 1.0880 to 4.1991 and a P-value of 0.0275.

Swallowing_Function_Score4: For this score, both in the original and imputed datasets, the relationship remains non-significant, evident from their high P-values (0.8172 and 0.9416 respectively).

Swallowing_Function_Score5: In the original data, despite an OR of 1.9167, its 95% CI ranges from 0.7743 to 4.7444 with a P-value of 0.1594, indicating a non-significant association. However, in the imputed data, an OR of 2.0390 corresponding to a P-value of 0.0464 and a 95% CI of 1.0115 to 4.1101 showcases a significant positive association.

Table 2 Odds Ratios and Confidence Intervals for Swallowing Function Scores: Comparison Between Original and Imputed Data

Dataset	Term	OR	CI_low	CI_high	P_Value
Original	Swallowing_Function_Score2	1.523	0.866	2.678	0.144
Original	Swallowing_Function_Score3	2.878	1.392	5.954	0.004
Original	Swallowing_Function_Score4	1.134	0.390	3.299	0.817
Original	Swallowing_Function_Score5	1.917	0.774	4.744	0.159
Imputed	Swallowing_Function_Score2	1.320	0.830	2.101	0.241
Imputed	Swallowing_Function_Score3	2.137	1.088	4.199	0.027
Imputed	Swallowing_Function_Score4	1.036	0.406	2.640	0.942
Imputed	Swallowing_Function_Score5	2.039	1.012	4.110	0.046

(3)Conclusion:

We believe that, through the above sensitivity analysis, we provide readers with a comprehensive, transparent view to evaluate our data processing method. Even though imputation has effects on certain variables (like Swallowing_Function_Score), we've taken the necessary steps to ensure the reliability and robustness of our analysis results.
