# Supplementary figures and images for: Machine learning-based prediction of symptomatic intracerebral hemorrhage after intravenous thrombolysis for stroke: a large multicenter study
Source: Front Neurol. 2023 Oct 20;14:1247492. doi: 10.3389/fneur.2023.1247492 (PMC10624225; doi:10.3389/fneur.2023.1247492)

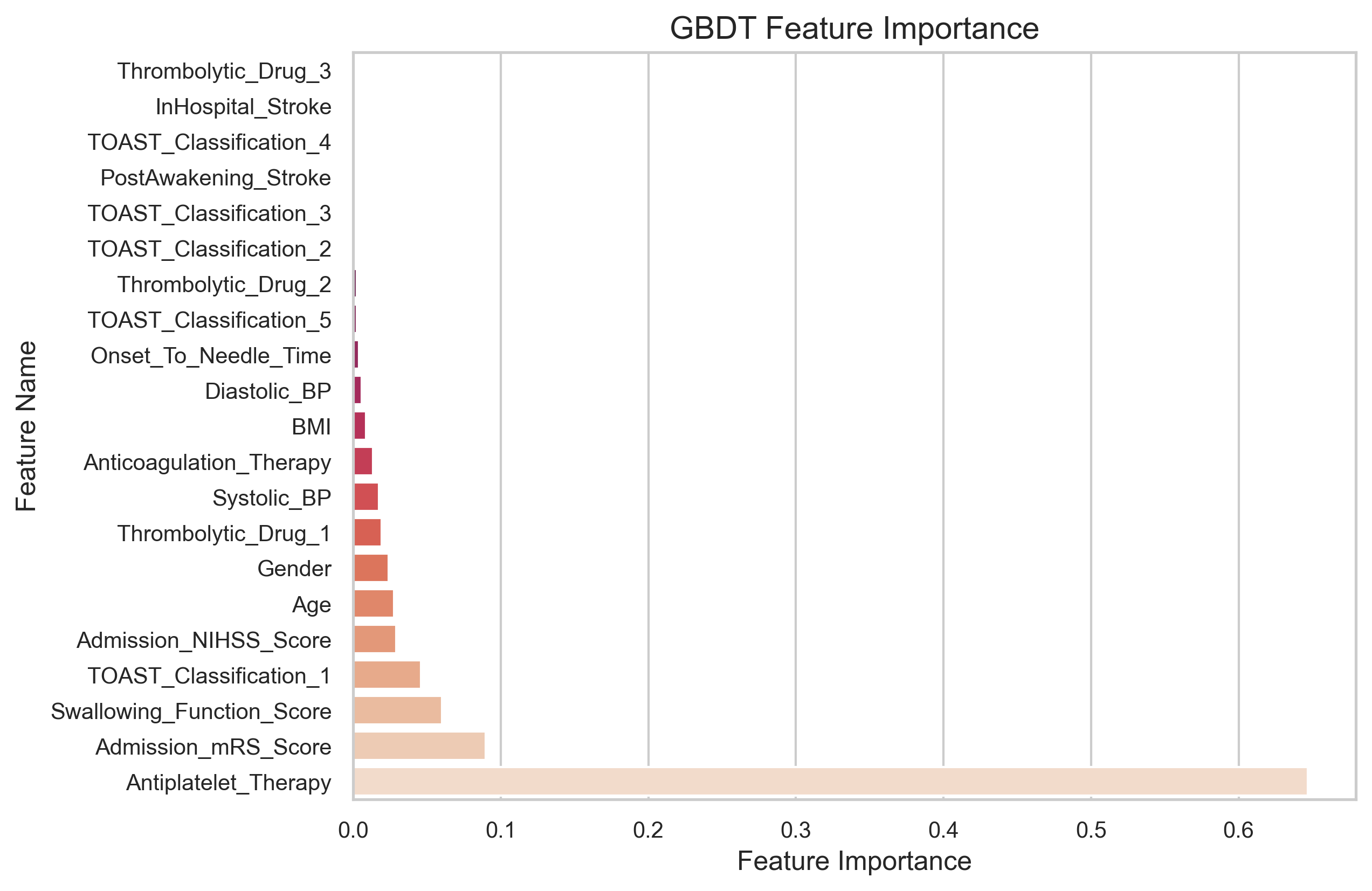

Supplement: Supplementary file 5 [file Image_1.PNG]

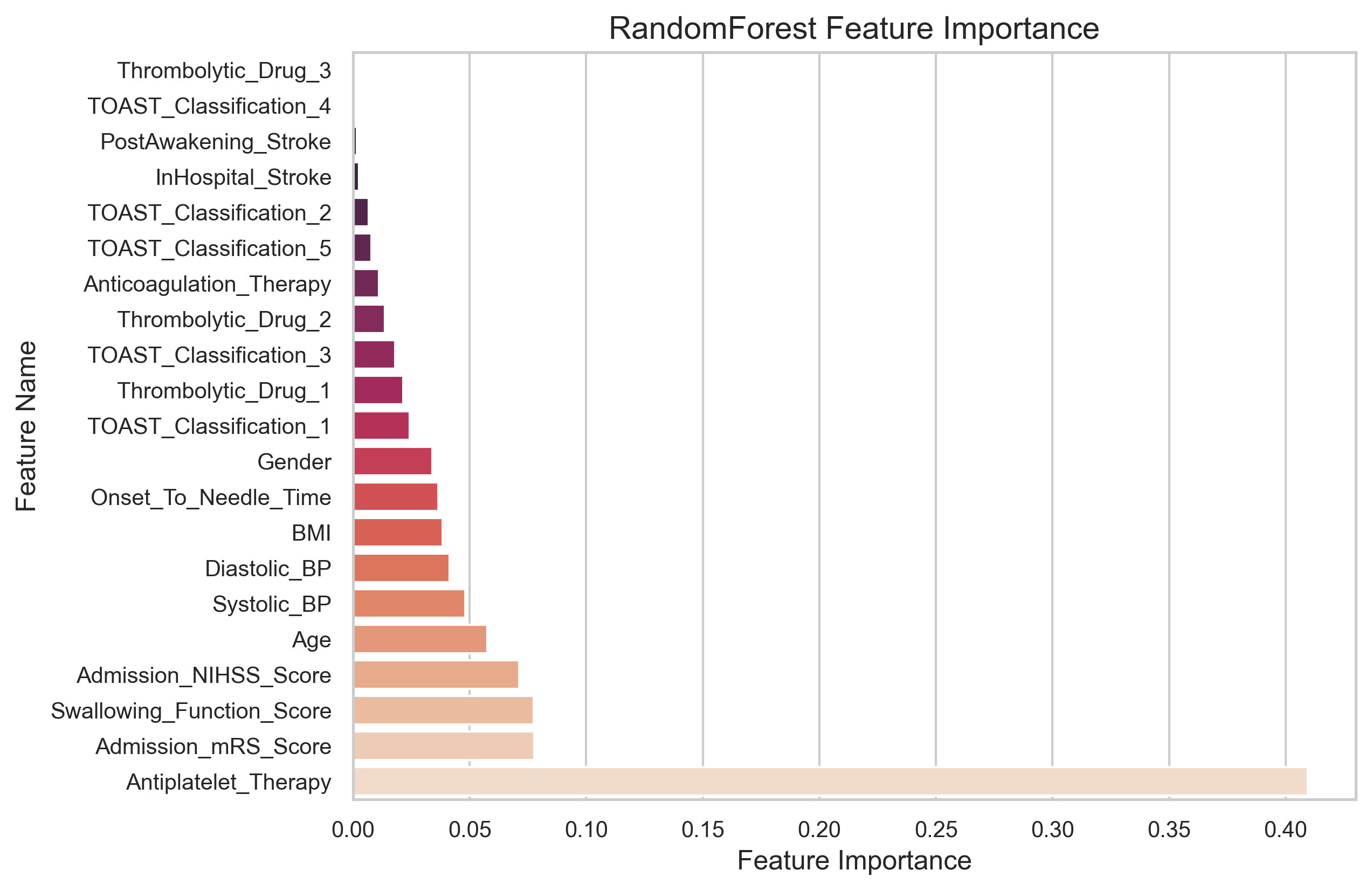

Supplement: Supplementary file 6 [file Image_2.PNG]
